# Are HPV Vaccines Well Accepted among Parents of Adolescent Girls in China? Trends, Obstacles, and Practical Implications for Further Interventions: A Five-Year Follow-Up Study

**DOI:** 10.3390/vaccines12091073

**Published:** 2024-09-19

**Authors:** Yu Huang, Jie Ling, Xiang Zhao, Qiaohong Lv, Lei Wang, Qingqing Wu, Shuiyang Xu, Xuehai Zhang

**Affiliations:** 1 Department of Health Education, Zhejiang Provincial Center for Disease Control and Prevention, 3399 Binsheng Road, Binjiang District, Hangzhou 310051, China; yhuang@cdc.zj.cn (Y.H.); xzhao@cdc.zj.cn (X.Z.); qhlv@cdc.zj.cn (Q.L.); leiwang@cdc.zj.cn (L.W.); qqwu@cdc.zj.cn (Q.W.); shyxu@cdc.zj.cn (S.X.); 2 Department of Health Education, Jiaxing Center for Disease Control And Prevention, Jiaxing 314050, China; lingjie_22@163.com

**Keywords:** HPV awareness, acceptance, HPV vaccine, Chinese adolescent girls, cervical cancer, parents, follow-up study

## Abstract

Background: In 2020, the WHO proposed the global strategic goal of accelerating the elimination of cervical cancer (CC). One of the key strategies is that, by 2030, 90% of girls will complete HPV vaccination by age 15. In 2017, HPV vaccines were first marketed in China. Objectives: This study aimed to explore the changes in parents’ knowledge about CC, HPV, HPV vaccines, and acceptance of HPV vaccination 5 years after the introduction of HPV vaccines into China. Associated factors and reasons for refusal by parents were also explored. Methods: A school-based follow-up study was conducted among parents in China from May 2018 to May 2023. Comparison using Chi-square tests was performed to measure the changes. Univariate and multivariate analyses were used to explore factors associated with HPV vaccination among teenage girls. Results: The overall cognitive level in terms of CC- and HPV-related knowledge among parents increased, and they expressed more willingness to vaccinate their daughter (35.4% in 2018 vs. 56.6% in 2023). The HPV vaccination rate among teenage girls remained at a low level (4.4% in 2018, 9.8% in 2023). The main obstacles reported by parents were limited knowledge (42.0%), scarcity of the HPV vaccine (29.2%), waiting until their daughter was older (27.5%), safety concerns (14.7%), high cost (9.0%), and effectiveness concerns (4.6%). Parents who are elderly, local residents, have received HPV vaccines themselves, have had experience of vaccinating their children with influenza vaccines, and have a higher knowledge level of CC, HPV and HPV vaccines are more likely to vaccinate their children with HPV vaccines. Conclusions: Although we observed an increment in parents’ knowledge level in terms of CC, HPV, HPV vaccines, and HPV vaccine uptake over the past 5 years, the HPV vaccine coverage among girls still falls short of the WHO’s 2030 target. Comprehensive intervention strategies, including tailored health education among adolescents and their parents, doctor recommendations, and providing financial subsidies or free HPV vaccines are needed in China.

## 1. Introduction

In recent years, the incidence rate and mortality of cervical cancer (CC) have remained high, and it has become one of the major malignant tumors that seriously threaten women’s health [1]. According to the data of the World Health Organization/International Agency for Research on Cancer (WHO/IARC), in 2020, the incidence rate and mortality of CC ranked fourth among female cancers worldwide, with about 604,000 new cases and 342,000 deaths, and the incidence rate and mortality rate were 13.3/100,000 and 7.3/100,000, respectively [2]. In 2020, the number of new cases of CC in China was about 109,700, and the number of deaths was about 59,000. The incidence rate and mortality rate were 10.7/100,000 and 5.3/100,000, respectively [3].

Research has revealed that HPV infection was present in 99.7% of CC cases [2]. In China, according to an epidemiological survey involving 1.7 million women in 2019, the HPV infection rate among Chinese women is 15.54% [4]. One of the important measures to reduce HPV infection and to eliminate CC is to vaccinate the target population against HPV. Globally, three types of HPV vaccines have been successfully developed and launched in 2006, 2007, and 2014, respectively [5]. They are quadrivalent vaccines targeting HPV 6, 11, 16, and 18, bivalent vaccines targeting HPV 16 and 18, and nine-valent vaccines targeting HPV 6, 11, 16, 18, 31, 33, 45, 52, and 58. A meta-analysis article published in The Lancet pointed out that countries implementing HPV vaccination programs for adolescent women have significantly reduced the incidence of HPV infection, cervical precancerous lesions (CIN2+), and anal genital warts. Vaccination is not only beneficial for young women but also for older women and men, as a result of herd immunity [6].

In November 2020, the WHO proposed the global strategic goal of accelerating the elimination of CC, hoping that countries can rely on policy guidance, national investment, and global joint efforts to embark on the path of eliminating CC. China promises to fully support the WHO’s strategy of accelerating the elimination of CC. To achieve the WHO’s goal, one of the most important strategy is that, by 2030, 90% of girls should be vaccinated against HPV by age 15 [1].

In China, bivalent, quadrivalent, and nine-valent HPV vaccines were approved for marketing in mainland China in July 2016, May 2017, and April 2018, respectively. In 2019, the first domestically produced HPV vaccine in China was approved for marketing [7]. These vaccines covered females aged from 9 to 45 years old [8,9]. Compared to Western countries, the HPV vaccine entered the Chinese market 10 years late, and millions of Chinese women have missed the opportunity to receive the HPV vaccine.

Vaccines in China are usually divided into two categories. Category I vaccines are usually provided for free and compulsorily by the government. Category II refers to other vaccines provided to recipients on demand and at their expense. The HPV vaccines are classified as Category II vaccines and not yet covered by the National Immunization Program. However, in some regions of China, domestic HPV vaccines are provided freely to school-aged girls.

Since entering the Chinese market, research on HPV vaccines has never ceased. A survey of over 4000 adult women from 30 provincial capital cities in China showed that three years after the approval of the HPV vaccine in China, only 3.1% of women aged 18–45 received the HPV vaccine [10]. Zhejiang is a highly developed province in eastern China. In 2018, a survey was conducted on mothers of schoolgirls in Zhejiang province, and it was found that only 54.3% of mothers knew that HPV vaccines could prevent CC, and only 4.4% had vaccinated their daughters [11]. Parents’ understanding and attitude toward CC and HPV vaccines are important influencing factors in terms of adolescents’ HPV vaccination [12,13,14,15,16]. In order to improve the vaccination coverage of HPV vaccines among adolescents, health education intervention has been carried out throughout Zhejiang in China since 2018.

We conducted a survey in 2023 among parents of girls from junior and senior high school about their awareness, attitudes toward CC and HPV vaccines, HPV vaccination status, and willingness to vaccinate their daughter, which we used to compare with data from five years ago. This study can provide scientific information for further promotion of the HPV vaccination program in China.

## 2. Materials and Methods

### 2.1. Study Design and Sample Size

This was a school-based follow-up study conducted from May 2018 to May 2023 in Zhejiang, China. In 2018, one year after the introduction of the HPV vaccine into China, we conducted our first survey to explore the knowledge of CC and HPV vaccine among parents of teenage girls, as well as the vaccination status of their daughters and willingness of parents to vaccinate their daughter in Zhejiang province. To track the changes over the past 5 years, we conducted a similar survey in 2023 and compared the relevant indicators with those from 5 years ago.

Estimating 50% CC awareness with a 10% allowable error, a non-response rate of 20%, and a 95% confidence interval (CI), we determined that a sample of 600 parents was required for our research. However, in order to facilitate sub-group comparisons, the total sample size was doubled, resulting in a final figure of 1200. In each school, we randomly selected 50 parents of girls for the study. In total, 12 junior high schools and 12 senior high schools were needed and randomly selected. An online survey questionnaire was distributed to those parents. In 2023, all the parents of girls in those schools were recruited for the study.

### 2.2. Data Collection and Quality Control

The questionnaire collected sociodemographic data, parents’ knowledge regarding CC, HPV, and HPV vaccines, HPV vaccination status in adolescents, parental HPV vaccination status, and adolescents’ influenza vaccination status. The questions about knowledge of CC were as follows: (1) “Is cervical cancer one of the most common genital-system cancers among women worldwide?” (2) “Do you agree that prevention of cervical cancer does not just concern older women?” (3) “Do you think HPV causes cervical cancer?”. The questions about awareness and knowledge of HPV and HPV vaccines were as follows: (1) “Is HPV primarily transmitted among humans through sex?” (2) “Are you aware that there are vaccines for cervical cancer?” (3) “Do you know the optimal age for HPV vaccination?”. The questions about behavior and attitude toward HPV vaccine and sex were as follows: (1) “Have you received HPV vaccination?” (2)“Have your children received HPV vaccination?” (3) “Have your children received influenza vaccination?”.

The questionnaire was hosted on wenjuan. Wenjuan (Changsha Ranxing Information Technology Co., Ltd., Changsha, China) which is a professional online questionnaire, test, assessment, and voting platform, focusing on providing users with powerful and humanized online design questionnaires, data collection, customized reports, survey results analysis and other series of services.

In order to control the quality of the survey, we set up a quality control question in the questionnaire to identify survey respondents who did not answer the questions seriously. The question was as follows: “This is a quality control question, please choose B.” All the survey subjects who chose other options were not included in the final data analysis.

### 2.3. Statistical Analysis

Data were exported from the wenjuan website to Excel (Microsoft, Redmond, WA, USA) and were analyzed using the Statistical Package for the Social Sciences (SPSS), version 19.0 (IBM Corporation, Armonk, NY, USA). Standard descriptive statistics were used for continuous and categorical variables. The Chi-square test was used to compare differences between study results from year 2018 and year 2023. Univariate and multivariate logistic regression analyses were used to explore the factors associated with HPV vaccination in adolescents. The factors included sociodemographic variables, influenza vaccination status, parental HPV vaccination status, parents’ knowledge related to CC and HPV vaccine. Odds ratios with 95% confidence intervals were used to express measures of the associations. *p* values of <0.05 were considered to represent significance (two-sided).

## 3. Results

### 3.1. Respondents’ Characteristics

**Figure 1:**
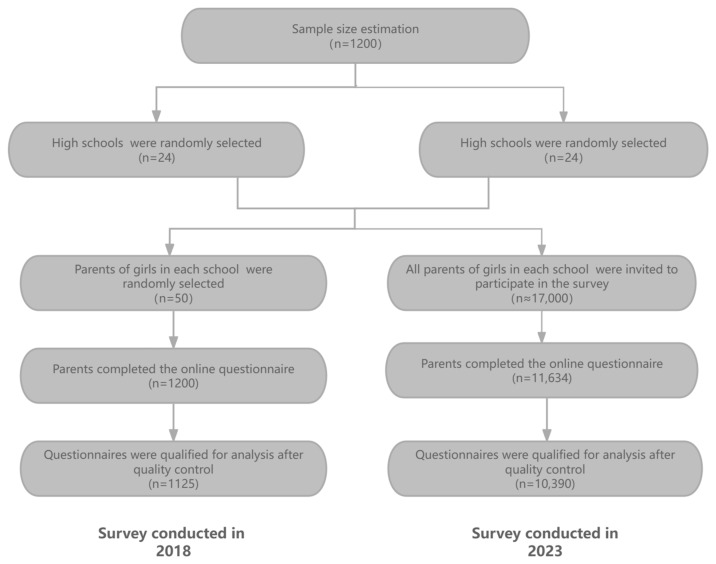
Flowchart of sample inclusion.

In the baseline survey, 1200 parents of girls were invited to participate, and 1125 people submitted qualifying survey questionnaires. In the 2023 survey, the questionnaire was distributed to the parents of all girls. Finally, 10,390 people submitted qualifying questionnaires and were included in the analysis (Figure 1).

In the survey conducted in 2023, women accounted for 88.4% (9182/10,390) and men accounted for 11.6% (1208/10,390). The majority of people were between 40 and 50 years old, accounting for 65.2% (6775/10,390). Compared with the survey conducted in 2008, the 2023 survey shared consistent sociodemographic characteristics except for the occupational composition. Refer to Table 1 for details.

### 3.2. Changes in Parents’ Knowledge of CC- and HPV-Related Information

Table 2 shows that the overall cognitive level in terms of CC- and HPV-related knowledge among parents in 2023 was higher than that in 2018. In 2018, the proportion of parents who agreed that CC is one of the most common genital-system cancers among women worldwide and of parents who agreed that the prevention of CC does not just concern older women were 87.5% and 47.1%, which increased to 92.4% and 61.4% in 2023, respectively. The difference was significant (*p* < 0.0001).

As for knowledge about HPV, 78.7% of parents knew that HPV causes CC, and 54.3% knew that HPV is primarily transmitted among humans through sex in 2018; in 2023, the proportion was 83.9% and 74.7%, respectively. The difference was significant (*p* < 0.0001).

As for HPV vaccine, we also found positive changes. The proportion of parents who knew that there are vaccines for CC and the optimal age for HPV vaccination increased from 54.3% and 35.6% to 62.9% and 49.7% from 2018 to 2023, respectively. More parents expressed willingness to vaccinate their children with the HPV vaccine (35.4% in 2018 vs. 56.6% in 2023). The difference was significant (*p* < 0.0001).

### 3.3. Changes in HPV Vaccination Coverage among Adolescent Girls

In 2018, we surveyed 1125 parents of girls from randomly selected junior high schools and senior high schools, where 4.4% (50/1125) of their children had received the HPV vaccine. In 2023, 9.8% (1020/10,390) had vaccinated their daughter with the HPV vaccine. The HPV vaccine uptake rate of high school students in 2023 was 2.23 times higher than that of students in 2018. This difference between the two groups was significant (Table 3).

### 3.4. Reasons for Not Receiving HPV Vaccine among Adolescent Girls

**Figure 2:**
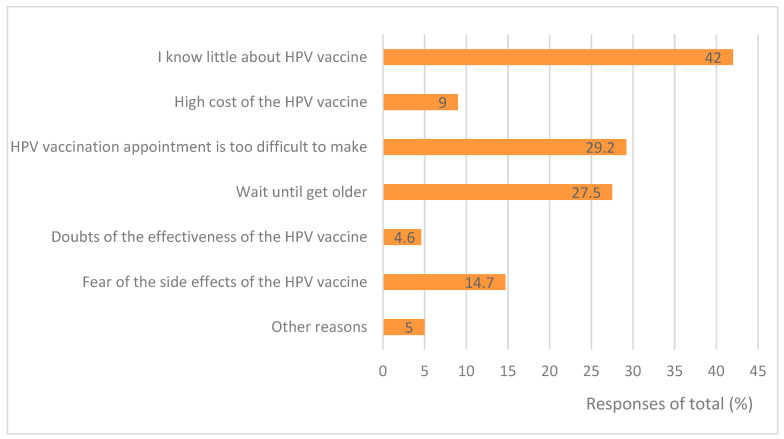
The main reasons why parents do not give their children HPV vaccination.

Figure 2 shows that, of the 9370 participants who did not vaccinate their daughter with the HPV vaccine, 3931 (42.0%) claimed that they knew little about the HPV vaccine, 2735 (29.2%) claimed the HPV vaccination appointment is too difficult to make, 2577 (27.5%) would like to wait until their daughter is older, 1377 (14.7%) feared the side effects of the HPV vaccine, 426 (4.6%) doubted the effectiveness of the vaccine, 839 (9.0%) complained of the high cost of the HPV vaccine, and 468 (5.0%) claimed other reasons for not wanting to their daughter to be vaccinated.

### 3.5. Factors Associated with Uptake of the HPV Vaccine among Adolescent Girls

As shown in Table 4, age, immigration status, teenagers’ influenza vaccination status, parental HPV vaccination status, knowing that there are vaccines for CC, and knowing the optimal age for HPV vaccination were statistically associated with teenagers’ uptake of the HPV vaccine. After controlling for other variables, the probability of receiving the HPV vaccine among teenagers whose parent were aged 40 to 50 and those over 50 was 1.6 times (AOR: 1.6, 95%CI: 1.3–1.9) and 1.7 times (AOR: 1.7, 95%CI: 1.3–2.2) higher than those under 40, respectively.

The probability of being vaccinated with the HPV vaccine among teenagers who receive the influenza vaccine every year (AOR: 2.1, 95%CI: 1.7–2.7) and those who receive the influenza vaccine some years (AOR: 1.3, 95%CI: 1.1–1.5) was higher than those who have never received the influenza vaccine. Teenagers who were local residents (AOR: 1.3, 95%CI: 1.1–1.6) and whose mothers have received the HPV vaccine (AOR: 6.2, 95%CI: 5.3–7.2) were more likely to be vaccinated when compared to their counterparts. The probability of being vaccinated with the HPV vaccine among teenagers whose parents were aware that there are vaccines for CC was 1.3 times (AOR: 1.3, 95%CI: 1.1–1.5) higher than their counterparts. Teenagers whose parents knew the optimal age for receiving the HPV vaccines were 2.9 times more likely to be vaccinated (AOR: 2.9, 95%CI: 2.5–3.4) when compared to those whose parents did not know it.

## 4. Discussion

### 4.1. Principle Findings

This study was conducted to assess the changes in parents’ knowledge about CC, HPV, HPV vaccines, and their willingness to vaccinate their daughter with the HPV vaccine five years after the introduction of HPV vaccines into mainland China. The results of our study showed a positive change. However, there is still a large proportion of parents who are not aware of the HPV vaccine and have doubts about its effectiveness and safety. The HPV vaccine coverage among adolescent girls in Zhejiang, China falls short of the WHO’s 2030 target.

This study found that the HPV vaccination rate among adolescent girls has increased from 4.4% in 2018 to 9.8% in 2023. However, over 90% of adolescent girls remain largely unprotected. According to the WHO’s recommendation, to achieve the goal of accelerating the elimination of CC, the following requirement shall be met by 2030: 90% of girls are vaccinated against HPV by the age of 15; 70% of women receive high-quality quantum CC screening once before age 35 and once before age 45; 90% of patients diagnosed with precancerous lesions and cancer receive standard treatment and management [1]. Thus, the current HPV vaccination rates among adolescent girls in Zhejiang in China fall well short of the WHO’s 2030 target. Recent studies conducted in Beijing (9.5%) and Guangzhou (3.1%) confirmed that low HPV vaccination rates are a common phenomenon in China [17,18]. The vaccination rate in China is much lower than that in developed countries. It was reported that in Scotland, the HPV vaccine uptake for at least one dose was 94.4% [19]. More than a 90% HPV vaccine uptake was also reported among girls (9–15 years) in the European Union [20]. The large gap may be explained by the fact that China started HPV vaccination more than 10 years later than those developed countries [8,9]. In addition, the COVID-19 pandemic may also have delayed the progress of the HPV vaccination program. Furthermore, in Australia and the UK, girls are routinely offered free HPV vaccination [21]. However, in China, HPV vaccines are more expensive compared to other vaccines. In our study, nearly 10% of parents cited the cost of the HPV vaccine as the reason for their reluctance.

Parents play a crucial role in deciding whether to have their child vaccinated against HPV. Consistent with previous research, the level of knowledge about HPV vaccines was found to be positively associated with parental willingness to accept them [22,23,24,25,26]. Our study revealed that as parents’ awareness of CC, HPV and HPV vaccines increased, so did their willingness to accept the vaccine. Specifically, we observed an increase in the percentage of parents who were aware of the threats posed by CC, understood that HPV causes CC, and knew how it is transmitted—from 87.5%, 78.7%, and 54.3% to 92.4%, 83.9%, and 74.7%, respectively. Regarding the HPV vaccine itself, there was also an increase in parental awareness of its existence for preventing CC and knowledge about the optimal age for vaccination—from 54.3%, 35.6% to 62.9% and 49.7%, respectively—resulting in a greater willingness among parents to vaccinate their children (35.4% in 2018 vs. 56.6% in 2023). Multivariate analysis confirmed these associations; girls whose parents were aware of the availability of vaccines for CC or knew the optimal age for receiving HPV vaccines had higher probabilities (AOR:1.3, 95%CI: 1.1–1.5; AOR:2.9, 95%CI: 2.5–3.4) of being vaccinated compared with their counterparts. This finding is probably explained by the data that parents with higher knowledge levels are more self-confident in decision-making.

In addition to parents’ knowledge of HPV vaccines, the parental vaccination status in terms of HPV vaccines was strongly associated with their daughters’ HPV vaccination. Teenagers whose mothers had received the HPV vaccine (AOR: 6.2, 95%CI: 5.3–7.2) were more likely to be vaccinated when compared to their counterparts. The positive association of vaccination status between parents and children was confirmed by other studies [27,28]. A study on the COVID-19 vaccine in China found that children were more likely to be unvaccinated if their parents did not take the COVID-19 vaccine. Among parents who had not taken the COVID-19 vaccine, 10.89% of their children were not vaccinated, whereas among parents who had taken COVID-19 vaccine, only 0.60% of their children were not vaccinated [27]. It can be explained that parents are the primary decision makers regarding obtaining vaccination for their child. If they administer a certain vaccine to themselves, it indicates that they trust that vaccine, which makes it a high possibility that they will vaccinate their children.

Age was also a factor that influenced children’s vaccination status. In our study, the probability of receiving the HPV vaccine among girls whose parents were aged over 50 was 1.7 times (AOR: 1.7, 95%CI: 1.3–2.2) higher than those under 40. This indicates that parents tend to vaccinate their daughter when they get older. In our study, 27.5% of parents claimed that they would like to wait until their daughter was older, which confirmed this indication.

In our study, there were several reasons reported by parents for why their daughter was not vaccinated with the HPV vaccine: limited knowledge of HPV, HPV vaccines, inadequate supplies of HPV vaccines, misguided safety concerns, high cost, etc. Over 40% of parents claimed that they know little about HPV vaccines, Nearly 30% of parents claimed the HPV vaccination appointment is too difficult to make, 27.5% of parents would like to wait until their daughter is older, 9.0% complained about the high cost, 14.7% feared side effects from the HPV vaccines, and 4.6% doubted its effectiveness. Results from studies conducted in other countries suggest similar reasons [25,29,30,31,32,33], although the proportions vary across different regions. A study carried out in Ethiopia found that the major reasons for not immunizing against HPV included scarcity/cost (57.4%), poor information (15.2%), doubts about vaccination (14.2%), and fear of side effects (7.6%) [25]. Another study conducted in Nigeria identified the high cost (55.6%), concerns about side effects (48.1%), and limited availability (25.9%) as the main factors [33]. Even European countries with high coverage face barriers due to insufficient information and safety concerns [13]. Research conducted five years ago in Zhejiang, China showed that the common reasons cited for not immunizing against HPV included fear of side effects (77.4%) and doubts about effectiveness (61.5%) [11]. The changes might be due to the possibility that as the number of vaccinated individuals increases, people’s doubts about the safety and effectiveness of vaccines may decrease, and a lack of knowledge about vaccines may become the main reason for not being vaccinated. The findings highlight an urgent need in China for school-based sexual health education on HPV infection and promotion of vaccination among adolescents and their parents—to capitalize on high levels of willingness toward achieving the WHO’s target by2030. Australia and China have successfully implemented school-based health education programs on HPV and HPV vaccines [34,35,36].

In addition to tailored health education, we suggest that government departments introduce encouraging policies, such as providing financial subsidies or free HPV vaccines, increasing vaccine production capacity to reduce HPV vaccine pricing, which will result in the greater affordability of HPV vaccines. China’s first domestically developed HPV vaccine, Cecolin, has obtained prequalification from the World Health Organization [37]. With its “superior quality” and “reasonable price”, this vaccine is expected to confer benefits upon a greater number of women globally, especially in developing countries [38]. Municipalities like Lianyungang in Jiangsu province and Jinan in Shandong province have already embarked on providing free domestic HPV vaccines to school-age girls [39]. To increase the acceptance of the HPV vaccine, it is highly recommended that healthcare providers in China inquire about individuals’ HPV vaccination status and educate girls and their parents regarding the merits of receiving the HPV vaccine during medical consultations. According to research conducted by Gerend et al., individuals who received advice from healthcare professionals to have an HPV vaccination were forty times more likely to do so [40], a finding corroborated by other studies as well [41,42,43,44].

### 4.2. Limitations

This study has certain limitations. Firstly, for the convenience of obtaining HPV vaccination-related information, we merely investigated the parents, thereby restricting the depth of the study. Secondly, the participants in the survey were parents of high school students in Zhejiang province, which might constrain the extent to which the results could be generalized to the entire nation. Thirdly, in these self-reported questionnaires, some outcomes that are susceptible to the influences of self-report bias should be interpreted with caution. Finally, parents may have multiple daughters but were only asked about their “daughter”, which may have a potential impact on the findings.

## 5. Conclusions

Although we observed an increment in parents’ knowledge level concerning CC, HPV, HPV vaccines, and an increasing willingness to vaccinate their daughter over the past 5 years, the HPV vaccine uptake among adolescents still falls short of the WHO’s 2030 target. Limited knowledge of HPV and HPV vaccines, inadequate supplies of HPV vaccines, misguided safety concerns, and high cost were common reasons reported by parents for not having their daughter vaccinated against HPV. Collaboration among schools, health workers, government, etc., is needed toward a successful HPV immunization program. It can be effective for schools to integrate education on HPV into the sexual health curriculum, for doctors to inform about the benefits of HPV vaccination when providing medical care, and for the government provide financial subsidies and promote domestically produced HPV vaccines to improve the affordability of HPV vaccines.

## Figures and Tables

**Table 1:** Sociodemographic characteristics of study participants.

Characteristic	Survey in 2018 n (%)	Survey in 2023 n (%)	*p* *
Gender			
Male	146 (13.0)	1208 (11.6)	0.181
Female	979 (87.0)	9182 (88.4)	
Age (years)			
<40	330 (29.3)	2753 (26.5)	0.082
40–50	714 (63.5)	6775 (65.2)	
50–60	81 (7.2)	862 (8.3)	
Education level			
Primary or lower (<9 years)	572 (50.8)	5008 (48.2)	0.234
Secondary (9–12 years)	302 (26.8)	2909 (28.0)	
Postsecondary (>12 years)	251 (22.3)	2473 (23.8)	
Occupation			
Government institution	152 (13.5)	1163 (11.2)	0.029
Business or service industry	523 (46.5)	4603 (44.3)	
Farmer	136 (12.1)	1403 (13.5)	
Stay-at-home mother/stay-at-home father/unemployed	153 (13.6)	1517 (14.6)	
Other (retired/business owner, etc.)	161 (14.3)	1704 (16.4)	
Annual household income (RMB)			
<100,000	626 (55.6)	5821 (56.0)	0.969
100,000–200,000	339 (30.1)	3100 (29.8)	
>200,000	160 (14.2)	1469 (14.2)	
Immigration status			
Resident	882 (78.4)	8208 (78.9)	0.635
Migrant from other counties of China	243 (21.6)	2182 (21.1)	
Marital status			
Married	1026 (91.2)	9487 (91.3)	0.902
Other (divorced/widowed/separated, etc.)	99 (8.8)	903 (8.7)	

* Derived from the Chi-square test.

**Table 2:** Cognitive changes in CC- and HPV-related knowledge among parents of high school students in Zhejiang, China, from 2018 to 2023.

Variables	Survey in 2018 n (%)	Survey in 2023 n (%)	χ^2^	*p* Value
Is cervical cancer one of the most common genital-system cancers among women worldwide?				
Yes	984 (87.5)	9602 (92.4)	33.52	<0.0001
No	141 (12.5)	788 (7.6)		
Do you agree that prevention of cervical cancer does not just concern older women?				
Yes	530 (47.1)	6378 (61.4)	86.18	<0.0001
No	595 (52.9)	4012 (38.6)		
Do you think HPV causes cervical cancer?				
Yes	885 (78.7)	8720 (83.9)	20.3	<0.0001
No	240 (21.3)	1670 (16.1)		
Is HPV primarily transmitted among humans through sex?				
Yes	779 (69.2)	7765 (74.7)	15.99	<0.0001
No	346 (30.8)	2625 (25.3)		
Are you aware that there are vaccines for cervical cancer?				
Yes	611 (54.3)	6532 (62.9)	31.56	<0.0001
No	514 (45.7)	3858 (37.1)		
Do you know the optimal age for HPV vaccination?				
Yes	400 (35.6)	5167 (49.7)	81.67	<0.0001
No	725 (64.4)	5223 (50.3)		
Would you approve of your children receiving HPV vaccination? *				
Yes	398 (35.4)	5881 (56.6)	184.43	<0.0001
No	727 (64.6)	4509 (43.4)		

* It included parents who had vaccinated their children with HPV vaccine.

**Table 3:** Changes in HPV vaccination among adolescent girls in Zhejiang, China, from 2018 to 2023.

Year	Number of Participants	Number of Participants Whose Children Received HPV Vaccine	HPV Vaccine Uptake (%)	χ^2^, *p* Value
2018	1125	50	4.4	χ^2^ = 34.76, *p* < 0.0001
2023	10,390	1020	9.8	

**Table 4:** Factors associated with HPV vaccine uptake among adolescents in 2023 in Zhejiang province.

Variables	HPV Vaccine Uptake (n, %)		OR (95%CI)	AOR (95%CI)
	Yes	No		
Gender of parents				
Female	845 (9.2)	8337 (90.8)	1	1
Male	120 (9.9)	1088 (90.1)	1.1 (0.9, 1.2)	1.0 (0.9, 1.1)
Age of parents (years)				
<40	153 (7.4)	1910 (92.6)	1	1
40–50	738 (10.3)	6410 (89.7)	1.4 (1.2, 1.7) *	1.6 (1.3, 1.9) ***
>50	129 (10.9)	1050 (89.1)	1.5 (1.2, 2.0) *	1.7 (1.3, 2.2) ***
Education level of parents				
Primary or lower (<9 years)	731 (9.7)	6794 (90.3)	1	1
Secondary (9–12 years)	191 (9.9)	1745 (90.1)	1.0 (0.9, 1.2)	0.9 (0.8, 1.1)
Postsecondary (>12 years)	98 (10.6)	831 (89.5)	1.1 (0.9, 1.4)	0.9 (0.7, 1.2)
Occupation of parents				
Government institution	46 (10.0)	414 (90.0)	1	1
Business or service industry	347 (9.6)	3281 (90.4)	1.0 (0.7, 1.3)	1.2 (0.8, 1.8)
Farmer	213 (11.4)	1650 (88.6)	1.2 (0.8, 1.6)	1.5 (0.9, 2.0)
Stay-at-home mother/stay-at-home father/unemployed	213 (9.5)	2032 (90.5)	0.9 (0.7, 1.3)	1.2 (0.4, 1.9)
Other (retired/business owner, etc.)	201 (9.2)	1993 (90.8)	0.9 (0.6, 1.3)	1.1 (0.7, 1.7)
Annual household income (RMB)				
<100,000	572 (9.8)	5249 (90.2)	1	1
100,000–200,000	288 (9.3)	2812 (90.7)	0.9 (0.8, 1.1)	1.0 (0.9, 1.2)
>200,000	160 (10.9)	1309 (89.1)	1.1 (0.9, 1.4)	1.2 (1.0, 1.5)
Immigration status				
Resident	884 (10.2)	7774 (89.8)	1.3 (1.1, 1.6) **	1.3 (1.1, 1.6) **
Migrant from other counties of China	136 (7.9)	1596 (92.1)	1	1
Influenza vaccin ation status				
Yearly	291 (18.5)	1280 (81.5)	3.2 (2.7, 3.9) ***	2.1 (1.7, 2.7) ***
Some years	543 (9.1)	5450 (90.9)	1.4 (1.2, 1.7) ***	1.3 (1.1, 1.5) ***
Never	186 (6.6)	2640 (93.4)	1	1
Marital status of parents				
Married	918 (9.7)	8569 (90.3)	1	1
Other (divorced/widowed/separated, etc.)	102 (11.3)	801 (88.7)	1.2 (1.0, 1.5)	1.2 (0.9, 1.5)
Parental HPV vaccin ation status				
Yes	382 (31.7)	824 (68.3)	6.2 (5.4, 7.2) ***	6.2 (5.3, 7.2) ***
No	638 (7.0)	8546 (93.0)	1	1
Is cervical cancer one of the most common genital-system cancers among women worldwide?				
Yes	931 (9.7)	8671 (90.3)	1	1
No	89 (11.3)	699 (88.7)	1.2 (0.9, 1.5)	1.2 (0.9, 1.5)
Do you agree that prevention of cervical cancer does not just concern older women?				
Yes	662 (10.4)	5716 (89.6)	1	1
No	358 (8.9)	3654 (91.1)	0.8 (0.7, 0.9) *	0.9 (0.7, 1.0)
Do you think HPV causes cervical cancer?				
Yes	865 (9.9)	7855 (90.1)	1	1
No	155 (9.3)	1515 (90.7)	0.9 (0.8, 1.1)	1.1 (0.9, 1.3)
Is HPV primarily transmitted among humans through sex?				
Yes	774 (10.0)	6991 (90.0)	1	1
No	246 (9.4)	2379 (90.6)	0.9 (0.8, 1.1)	1.1 (0.9, 1.4)
Are you aware that there are vaccines for cervical cancer?				
Yes	758 (11.6)	5774 (88.4)	1.4 (1.2, 1.6) ***	1.3 (1.1, 1.5) ***
No	340 (8.8)	3518 (91.2)	1	1
Do you know the optimal age for HPV vaccination?				
Yes	344 (20.1)	1366 (79.9)	3.0 (2.6, 3.4) ***	2.9 (2.5, 3.4) ***
No	676 (7.8)	8004 (92.2)	1	1

* *p* value < 0.05, ** *p* value < 0.01, *** *p* value < 0.001 and 1 = reference. OR = odds ratio; AOR = adjusted odds ratio; 95%CI = 95% confidence interval.

## Data Availability

The data presented in this study are available on request from the corresponding author. The data are not publicly available due to ethical restrictions.

## Abbreviations

HPV	human papillomavirus
CC	cervical cancer
OR	odds ratio
AOR	adjusted odds ratio
95%CI	95% confidence interval
